# Occupation Coding of Job Titles: Iterative Development of an Automated Coding Algorithm for the Canadian National Occupation Classification (ACA-NOC)

**DOI:** 10.2196/16422

**Published:** 2020-08-05

**Authors:** Hongchang Bao, Christopher J O Baker, Anil Adisesh

**Affiliations:** 1 Department of Computer Science Faculty of Science, Applied Science and Engineering University of New Brunswick Saint John, NB Canada; 2 Department of Computing Science University of Alberta Edmonton, AB Canada; 3 IPSNP Computing Inc Saint John, NB Canada; 4 Division of Occupational Medicine Department of Medicine University of Toronto Toronto, ON Canada; 5 Division of Occupational Medicine St Michael’s Hospital Toronto, ON Canada; 6 Faculty of Business University of New Brunswick Saint John, NB Canada

**Keywords:** occupation coding, automated coding, occupational health, job title

## Abstract

**Background:**

In many research studies, the identification of social determinants is an important activity, in particular, information about occupations is frequently added to existing patient data. Such information is usually solicited during interviews with open-ended questions such as “What is your job?” and “What industry sector do you work in?” Before being able to use this information for further analysis, the responses need to be categorized using a coding system, such as the Canadian National Occupational Classification (NOC). Manual coding is the usual method, which is a time-consuming and error-prone activity, suitable for automation.

**Objective:**

This study aims to facilitate automated coding by introducing a rigorous algorithm that will be able to identify the NOC (2016) codes using only job title and industry information as input. Using manually coded data sets, we sought to benchmark and iteratively improve the performance of the algorithm.

**Methods:**

We developed the ACA-NOC algorithm based on the NOC (2016), which allowed users to match NOC codes with job and industry titles. We employed several different search strategies in the ACA-NOC algorithm to find the best match, including exact search, minor exact search, like search, near (same order) search, near (different order) search, any search, and weak match search. In addition, a filtering step based on the hierarchical structure of the NOC data was applied to the algorithm to select the best matching codes.

**Results:**

The ACA-NOC was applied to over 500 manually coded job and industry titles. The accuracy rate at the four-digit NOC code level was 58.7% (332/566) and improved when broader job categories were considered (65.0% at the three-digit NOC code level, 72.3% at the two-digit NOC code level, and 81.6% at the one-digit NOC code level).

**Conclusions:**

The ACA-NOC is a rigorous algorithm for automatically coding the Canadian NOC system and has been evaluated using real-world data. It allows researchers to code moderate-sized data sets with occupation in a timely and cost-efficient manner such that further analytics are possible. Initial assessments indicate that it has state-of-the-art performance and is readily extensible upon further benchmarking on larger data sets.

## Introduction

In many research studies, and for governmental or other statistical purposes, data collection includes gathering information on occupation. Occupation is a widely used explanatory variable in health research, representing social status and class as well as exposure to environmental hazards [1]. Typically, such data are collected either by a self-completed questionnaire or by an interviewer. In either case, to be useful for secondary reuse in health care analytics, a coding system is applied to translate the job titles into meaningful categories and then match them to the appropriate standard codes. Manual coding is generally considered to be the most reliable approach; however, manual coding is very expensive and time-consuming and requires considerable expertise. It can, from our experience, take a manual coder 3-5 days to code 500 job titles.

The Canadian National Occupational Classification (NOC) 2016 is a 4-tiered hierarchical arrangement of occupational groups with successive levels of disaggregation [2]. It has 500 predefined occupational codes and unit groups that code >30,000 occupational job titles, although different unit groups may contain similar job titles. Occupational coding is therefore a complex task, with the possibility of different professional coders coding differently, sometimes developing individual coding preferences [3,4]. The agreement of manual occupation coders has been reported to range from 44% to 89% at the four-digit level [4]. Efforts have been made to automate such coding. Gweon [3] reported that researchers have implemented 2 kinds of automated coding approaches: a data-based approach and a rule-based approach.

A data-based approach involves using machine learning algorithms to create models from manually coded training data. Once the model has been trained, new data can be coded automatically [3]. Several different algorithms have been used for occupation coding in this manner. For example, Bethmann et al [5] employed 2 machine learning algorithms (Naive Bayes and Bayesian Multinomial) for occupation coding using a data set of 300,000 coded answers from the German National Educational Panel study. The approach used by Bethmann et al [5] was further deployed by Schierholz et al [6] to code 2 survey data sets from the German Institute for Employment Research. Gweon [3] proposed 3 methods for automatic coding: (1) combining separate models for detailed occupation codes into aggregate occupation codes, (2) a hybrid method that combines the duplicate-based approach with a machine learning algorithm, and (3) a modified nearest neighbor approach. These same authors [3] used data from the German General Social Survey (ALLBUS). In the United States, Russ et al [7] developed a stacked ensemble algorithm trained with 14,983 manually coded job titles to assign US SOC (Standard Occupational Classification) 2010 occupation codes based on job title, task, and industry input data. Nahoomi [8] used 65,962 SOC-coded job titles to report that support vector machines (SVMs) and convolutional neural networks (CNNs) have similar performance but perform better than Naive Bayes.

A rule-based approach involves building a classification algorithm based on several rules created by experts after analyzing manually coded data. Depending on their design, the application of rules can lead to the assignment of multiple classes to an input and a filter algorithm is applied to identify the best match. There exist several rule-based methods for automated matching of text in a hierarchical classification system. In one approach, an initial match between the input data and the text description of a top-level class triggers further matchmaking at lower levels in the hierarchy. Another method is to directly compare the input data with each of the hierarchical levels [9]. In the same study, Burstyn et al [9] applied an algorithm that mixes both methods to code against the SOC (2010) system. Schierholz [6] has since reported that this approach rarely coded >50% of records accurately.

A review of automated occupation coding system performance carried out by Nahoomi [8] considered the full range of approaches, including machine learning, hybrid, and rule-based approaches. It was found that occupation classification algorithms can deliver production rates of up to 100%, with accuracy levels ranging from 44% to 98%. The best dictionary-based approach produced a 43% production rate but a 94% accuracy, whereas machine learning approaches were able to deliver a 100% production rate and a maximum accuracy of 80%. The hybrid machine learning/rule-based approaches had a production rate of 73% to 80% and an accuracy rate of 98% to 100%, relying on large data sets and >3000 rules. Overall, the accuracy depends on the type of algorithms used and the manner in which accuracy is computed. The review did not identify any systems targeted or tested on the Canadian NOC.

In this study, we report on the development of a hybrid algorithm that uses a rule-based method for search and matchmaking and a filtering step to select the best match. The algorithm is rigorous in matching input text with the hierarchy textual descriptions of the NOC, meaning it is designed to accommodate poor-quality input data, specifically spelling and typing errors. It adjusts for inserted symbols such as hyphenation, forward slashes, or punctuation errors in the input strings. Multiple search strategies are performed sequentially until job titles are matched and filtered and the best-fitting NOC code is identified. This paper aimed to document the incremental design of the algorithm based on performance benchmarking and an analysis of uncoded or incorrectly coded occupation data.

## Methods

### Data Resources

The Canadian NOC is the national reference for occupations in Canada, providing a standard taxonomy and a Canadian framework for collecting, analyzing, and disseminating occupational data for labor market information and employment-related program administration. It comprises >30,000 occupational titles gathered into 500 unit groups, organized according to 4 skill levels and 10 skill types. Unit groups are based on the similarity of skills, defined primarily by functions and employment requirements. Each unit group describes the main duties and employment requirements and details examples of occupational titles. Each unit group has a unique four-digit code. The first 3 digits of this code indicate the major and minor groups to which the unit group belongs [2].

Table 1 lists the first level of NOC (2016). NOC (2016) is organized in a four-level hierarchy, and there are 10 broad occupational categories in the first level, 46 major groups in the second level, 140 minor groups in the third level, and 500 unit groups in the fourth level [2]. NOC major group 00: senior management occupations showing minor and unit groups are shown in Textbox 1.

**Table 1:** Occupational categories of the Canadian National Occupational Classification (first level).

NOC^a^ code	Occupational categories
0	Management occupations
1	Business, finance, and administration occupations
2	Natural and applied sciences and related occupations
3	Health occupations
4	Occupations in education, law and social, community, and government services
5	Occupations in art, culture, recreation, and sport
6	Sales and service occupations
7	Trades, transport, and equipment operators and related occupations
8	Natural resources, agriculture, and related production occupations
9	Occupations in manufacturing and utilities

^a^NOC: National Occupational Classification.

**National Occupational Classification major group 00: senior management occupations showing minor and unit groups.**

- 0 - management occupations
- 00 - senior management occupations
- 001 - legislators and senior management
- 0011 - legislators
- 0012 - senior government managers and officials
- 0013 - senior managers: financial, communications, and other business services
- 0014 - senior managers: health, education, social and community services, and membership organizations
- 0015 - senior managers: trade, broadcasting, and other services, n.e.c (not elsewhere classified)
- 0016 - senior managers: construction, transportation, production, and utilities

Multiple versions (2001, 2006, 2011, and 2016) of the NOC can be accessed through a web browser [10]. StatCan also provides programmatic access to the 2016 classification via web services including GetNOCStructure, GetSkillType, GetSkillLevel, GetMajorGroup, GetMinorGroup, GetUnitGroup, GetNOCTitles, GetNOCTitlesAutoCoding, GetNOCDescription, and GetNOCDescriptionByKeyword. Furthermore, the NOC database dump web method, DumpNOCDatabase, was available, enabling local query access to the categories.

#### Data Set

The initial data set used to benchmark the algorithm consisted of 566 job and industry titles with manual coding to the NOC (2016) and to the North American Industrial Classification (NAICS), respectively. These data were gathered as part of the Canadian Immunization Research Network Community Acquired Pneumonia study [11] to investigate occupational associations. The data were coded by 1 researcher and underwent validation checks by a second coder. Table 2 lists the input data set of this project that contains the fields *Current Job Title*, *NOC Code Manually*, and *Current Industry*. The *NOC Code Manually* was manually coded and entered based on the current job title and industry. The data on job title and industry originate from free text entered in response to the questions “What is your job title?” and “In which type of industry do you work?” with additional questions asking about the last job held for those not currently working and enquiry about the longest held post.

**Table 2:** Example of the source data sets.

Current job titles	NOC^a^ codes manually	Current industries
Owner of cleaning business	0651	Service industry
Managing website	0213	Computer technology
Manager	0311	Health care

^a^NOC: National Occupational Classification.

The current job title and the current industry set are accessed from the data set, and the algorithm interrogates the local version of the NOC database to find the best matching NOC code. The NOC code in the data set is contrasted with the algorithmically derived code to arrive at a performance evaluation.

### Algorithm

**Figure 1:**
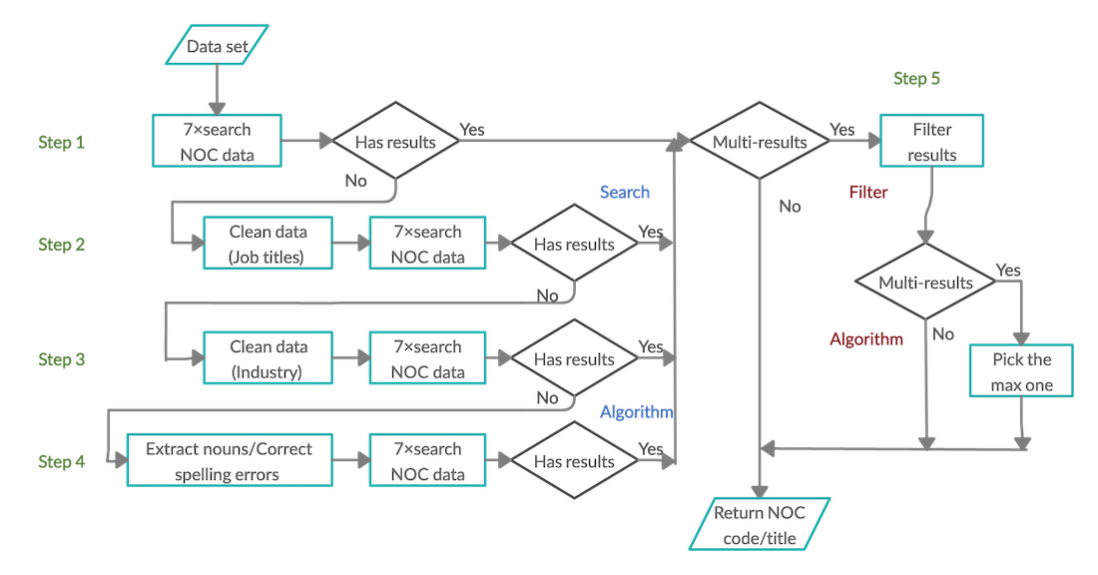
ACA-NOC algorithm. NOC: National Occupational Classification.

To develop an algorithm for automated occupation coding, direct access to the full NOC structure is required. Although manual coding relies on familiarization with a coding structure and computer-assisted keyword lookups through a web browser, we sought to use the NOC’s web service access to look up codes for our sample data. In preliminary work, we assessed the accuracy with which we could identify the correct job codes using individual web service calls to the job title field only. Albeit limited, we recorded this as a baseline for subsequent performance comparisons. To improve the accuracy beyond using the web services, we initiated the design of the Automated Coding Algorithm (ACA)-NOC algorithm. To facilitate custom searches, the NOC data were downloaded and saved in a spreadsheet using the *NOC Database Dump Web Methods* provided by NOC. Thereafter, we designed a search algorithm, as shown in Figure 1, employing the 7 search strategies listed in the section *Search Strategies* to match text input strings with NOC titles and text descriptions in the locally stored data.

#### Search Algorithm

The search algorithm shown in Figure 1 comprises a sequence of steps that iteratively look for matches in the NOC until all the given job titles in the input data set are matched with one or more NOC codes. The initial step, step 1, runs multiple search strategies sequentially to match input data with the NOC data using either a job title or an industry description. Depending on the outcomes from these NOC data searches, matches to a single NOC code are archived or multiple NOC code matches are further filtered according to the NOC hierarchy and by industry, step 5. If no matches are generated, the input data are preprocessed (splitting job titles, removing stop words, stemming, and noun extraction from titles) in step 2 before NOC multisearches are reinitiated. If no NOC codes are matched at this stage, the input industry descriptions are preprocessed and NOC searches are reinitiated, step 3. In step 4, any input data that still fail to link to an NOC code are further processed to extract any nouns in the job title, and spelling checks and corrections are made. This is the final step in which NOC data searches are subsequently reinitiated.

#### ACA-NOC Algorithm

We applied multiple search strategies sequentially to match the input data with the NOC data.

1. Query the NOC job codes by exactly matching the input data with the NOC job titles in step 1.
2. If results are found, then initiate postprocessing in step 5. If not, then use the following procedure:
  - 2.1. Obtain results from the processed information (splitting job titles, removing stop words, stemming, and noun extraction from titles) in step 2.
  - 2.2. If results are found, then perform postprocessing in step 5. If not:
    - 2.2.1. Query NOC information with industry or split industry keywords in step 3.
    - 2.2.2. If results are found, then perform postprocessing in step 5. If not:
      - 2.2.2.1. Correct spelling errors for the input data. Then, query NOC data in step 2.2.2.2. If results are found, perform postprocessing in step 5. Otherwise, return none.
      - 2.2.2.2. If results are found, perform postprocessing in step 5. Otherwise, return none.
3. Postprocessing:
  - 3.1. Return the NOC code if a single result is found, else:
  - 3.2. Apply the filter algorithm to the multiple results and return the most pertinent NOC code in step 5.

#### Search Strategies

1. Exact: this search returns results that exactly match the input string(s) entered.
2. Minor exact: this search returns results that match exactly after correcting spelling errors in the input string(s).
3. Like: this search returns results that include every input string in the specific order as entered. The exact string may be included anywhere in the associated text.
4. Near (same order): this search returns results that include every input string matched in the same order.
5. Near (different order): this search returns results that include every input string matched in any order.
6. Any: this search returns results that include any/some of the string(s) entered.
7. Weak match: this search returns results that include any of the nouns found in the string(s) entered.

#### Filter Algorithm

The filter algorithm selects a single NOC code from a list of candidate NOC codes based on the frequency of the keyword (job title or industry title) in the NOC descriptions. The selection occurs in the following order:

1. Select the given job titles from the NOC skill-type names in the result sets.
2. Select the given job titles from the NOC major group names in the result sets.
3. Select the given job titles from the NOC minor group names in the result sets.
4. Select the given job titles from the NOC group titles in the result sets.
5. Select the industry title from the NOC job titles in the result sets.
6. Split the industry titles that have inserted symbols and search each of the job titles from the NOC job titles in the result sets. Then, return the data set with the highest frequency of the keyword.
7. Select the industry title from the NOC descriptions (lead statement, main duties, and employment requirements) in the result sets.

## Results

### Overview

The design of the ACA-NOC algorithm shown in Figure 1 was arrived at through an iterative process of design, deployment, and testing. Each generation of the algorithm was evaluated using the sample data set described in the section *Data Set*. In this section, we detail the different generations of the algorithm and the performance at each generation. Production rate and accuracy are metrics that were used for evaluating the performance, where the production rate is the proportion of observations that can be coded automatically. For a given production rate, accuracy is the proportion of codes that are coded correctly when compared with manual coding [3]. Accuracy can be assessed for each digit of the NOC code.

The ACA-NOC algorithm underwent 4 generations of design and evaluation. Table 3 lists the production rate and accuracy of each version of the algorithm compared with the simple web service lookup.

The first observation that could be made was that ACA-NOC generation 0 outperformed the web service on production rate, and a 100% production rate was achieved in ACA-NOC generation 1. Second, between ACA-NOC generation 0 and ACA-NOC generation 4, the accuracy in coding the NOC skill-type level (a one-digit code) improved from 67.3% to 73.3%, the NOC major group level (two-digit code) improved from 58.3% to 64.0%, the NOC minor group level (three-digit code) improved from 53.5% to 59.7%, and the NOC unit group level (four-digit code) improved from 51.2% to 55.5%. Expert review by a team member (author AA) familiar with occupational coding aided the development of the algorithm through identification of mismatched items and discussion within the development team of the likely reasons for error. These discussions led to modifications of the algorithm and further improved the performance. Finally, the expert review allowed the recognition that a number of mismatches did not necessarily indicate poor performance by the algorithm, but that, in some cases, the algorithm had identified a more accurate code. Specifically, human error caused data entry errors, and the algorithm code and manual code were both reasonable choices.

**Table 3:** Production rate and accuracy of occupation coding (NOC 2016) with the ACA-NOC algorithm by generation number (N=566).

Generation numbers	Production rate, %	Accuracy, %			
		1 digit	2 digits	3 digits	4 digits
Web service^a^	74.0	61.3	52.8	49.3	47.2
0	93.5	67.4	58.4	53.6	51.2
1	100	69.3	60.4	55.8	53.0
2	100	70.3	61.3	56.5	53.9
3	100	70.5	61.8	57.2	54.6
4	100	73.3	64.0	59.7	55.5
4 (following expert review of mismatches)	100	81.6	72.3	65.0	58.7
Mannetje and Kromhout [4] agreement rates (%) for occupation classification between manual coders	N/A^b^	75-97	61-92	56-80	44-89

^a^The method we used was *GetNOCTitlesAutoCoding*. This method returned a best match NOC code for the input titles. It queried the database titles tables first; if no result was found, then it queried database profile tables. The important parameters of the method are strTitle->job title, bytLang->0 (english), bytMultiTitles->0 (not required), bytGrouptitle->0 (not required).

^b^N/A: not applicable.

### Generation 0

The first version of the algorithm included only steps 1 to 3. No hierarchical filtering steps were applied.

1. Step 1: if the entry of *Current Job Title* exists, then search for NOC job titles using the same. Otherwise, search for NOC job titles by industry keywords. If the search returns only 1 NOC code, then the search for this job title is completed.
2. Step 2: if the returned data set is *none*, then remove stop words or stem the job titles and search the NOC job titles using the modified job titles. If there are no results, search NOC descriptions using the job title provided. If the search returns only 1 NOC code, then the search is completed. If it returns >1 NOC code, proceed to step 3.
3. Step 3: if the returned data set contains >1 NOC code, search the returned data set using industry keywords. If there are no results, then search the lead statement related to the returned data set using industry keywords. Rank the results in alphabetical order and return the last one.

#### Search Strategies

1. Exact: this search returns results that exactly match the string(s) entered.
2. Like: this search returns results that include every string in the specific order as entered. The string may be included anywhere in the associated text.
3. Near: this search returns results that include every string entered in any order.
4. Any: this search returns results that include any of the strings entered.

#### Performance

The initial data set consisted of 566 job and industry titles with manual coding to the NOC (2016) and to the NAICS, respectively. Applying the ACA-NOC algorithm in its original form, Generation 0, to the input data set from step 1 to step 3 assigned NOC codes to 529 cases (93.5%) and resulted in 37 (6.5%) residual uncoded job titles. For 289 (51.2%) job titles, the assigned NOC codes matched the manual coding NOC codes at the fourth digit position, that is, identical matches to the manual coding NOC codes; for 303 (53.6%) job titles, the assigned NOC codes matched the manual coding NOC codes at the third digit position; for 330 (58.4%) job titles, the assigned NOC codes matched the manual coding NOC codes at the second digit position; for 381 (67.4%) job titles, the assigned NOC codes matched the manual coding NOC codes at the first digit position; and for 148 (26.2%) job titles, the assigned NOC codes differed from the manual coding NOC codes, with no matches on the 4 digits of the NOC code.

### Generation 1

Following the evaluation of Generation 0, a review of cases in which the algorithm failed to code input job titles and industry descriptions was made. Table 4 lists cases showing that some current job titles contain a forward slash, such as *Interpretor/Translator*; some have spelling errors, such as *Constuction*; and some contain adjectives and spelling/typographical errors, such as *certified accontant*.

Consequently, the algorithm was updated as follows: any unmatched job titles are reviewed for (1) incorrect spelling. Correct the spelling errors before continuing NOC searches with the corrected job titles. (2) Inserted symbols such as hyphenation, forward slash, or punctuation in the job titles initiated a subroutine where job titles were split and coded separately to the NOC. (3) Selected target nouns were extracted and used to search the NOC data. (4) Any nouns found in the job titles were extracted and used to search the NOC data.

**Table 4:** Examples of unmatched data for Generation 0.

Current job titles	NOC^a^ code by ACA^b^-NOC	NOC codes manually	Current industries
Interpretor/translator	N/A^c^	5121	N/A
CONSTUCTION	N/A	7611	CONSTUCTION
Certified accontant	N/A	1111	N/A

^a^NOC: National Occupational Classification.

^b^ACA: Automated Coding Algorithm.

^c^N/A: not applicable.

#### Performance

Compared with Generation 0, the improvements yielded 10 more job titles (58+20+5+5-48-18-9-3) accurately coded in the fourth position, 12 (4+2+1+3-2-1-5+10) more job titles accurately coded in the third position, 11 (4+2+9-6-4-1-5+12) more job titles accurately coded in second position, and 10 (8+4+1+18-8-2-2-20+11) more job titles accurately coded in the first position; all the current job titles matched with an NOC code. Table 5 lists the specific details of the improvement between the results of the previous Generation 0 and Generation 1 of the algorithm. Overall, 88 more job titles were accurately coded in the fourth position, although 78 job titles previously accurately coded in this position were lost. The reason for the lost matches was that these job titles were listed in alphabetical order where >1 job title was matched.

An example of an exact match that did not correlate with the manual code is the job title *Landscaper* in the industry *Landscaping*, where the algorithm assigned code 2225. This gave only a two-digit match with the manually assigned code 2215, which does not exist on investigation and represents a typographical source data input error. Therefore, the algorithm code was correct in finding the category *Landscape and horticulture technicians and specialists*, which includes the job title *landscaper*.

**Table 5:** Performance of Generation 1 compared with Generation 0.

G1^a^	G0^b^				
	No match	One-digit match	Two-digit match	Three-digit match	Four-digit match
No match	N/A^c^	8	4	4	58
One-digit match	8	N/A	2	2	20
Two-digit match	6	4	N/A	1	5
Three-digit match	2	1	0	N/A	5
Four-digit match	48	18	9	3	N/A

^a^G1: Generation 1.

^b^G0: Generation 0.

^c^N/A: not applicable.

### Generation 2

Table 6 lists the unmatched sample data from Generation 0 of the algorithm. It was identified that some of the industry titles also contained forward slash, punctuation, and hyphenation. Additionally, it was noted that some current job titles would match with the correct NOC code if only the noun from the title was used for matching. For example, a match to an NOC code was possible with *producer* but not *agricultural producer*.

The algorithm was rewritten to include the following updates designed to address these issues:

The correction of spelling errors was moved to the end of the algorithm. Industry titles including hyphenation, forward slash, or punctuation were split, and both parts of the titles were used to search for NOC data. Compound words with spelling errors were corrected.

The description of the ACA-NOC Generation 2 algorithm is as follows:

1. Step 1: is the same as the original algorithm.
2. Step 2: if the returned data set is *none*, search the NOC data based on job titles after each of the operations on the input data set, as follows:
  - 2.1 Split job titles.
  - 2.2 Remove stopping words.
  - 2.3 Stem job titles
  - 2.4 Extract nouns from job titles.
  If the search returns only 1 NOC code, then finish the search; if it returns >1 NOC code, then repeat step 5.
3. Step 3: if the returned data set is still *none*, search the NOC data based on industry. If there are no results, then the industry is split and searched again. If the search returns only 1 NOC code, then finish the search; if it returns >1 NOC code, repeat step 5.
4. Step 4: If the returning data set is still *none*, search the NOC data based on any nouns from the job titles. If there are no results, then correct spellings and search again. If the search returns only 1 NOC code, then finish the search; if it returns >1 NOC code, repeat step 5. If there are still no results, then return *none*.
5. Step 5: is the same as the original algorithm.

**Table 6:** Unmatched data for Generation 1.

Current job titles	NOC^a^ codes by ACA^b^-NOC	NOC codes manually	Current industries
MARKETING IN MD OFFICE	2153	1123	MARKETING/HEALTH CARE
Property manager	0714	1224	Real estate/housing
Agricultural producer	8252	0821	Farm

^a^NOC: National Occupational Classification.

^b^ACA: Automated Coding Algorithm.

#### Performance

Compared with Generation 1 of the ACA-NOC algorithm in Table 7, the improvements yielded 5 (10+2+0+1-5-2-1-0) more job titles accurately coded in the fourth digit position, 4 (0+1+1+0-1-1-0-1+5) more job titles accurately coded in the third digit position, 5 (2+0+0+1-1-0-1-0+4) more job titles accurately coded in the second digit position, and 6 (4+0+1+2-3-0-1-2+5) more job titles accurately coded in the first digit position. A total of 13 additional job titles were accurately coded in the fourth digit position, but 8 job titles accurately coded in this position were lost because of the alphabetical selection order for multiple matches.

On reviewing apparent mismatches, the algorithm coded the job title *Addictions worker* with industry *hospital* to 4212 *Social and community service workers*, which gave a single digit match with the manually assigned code 4153 *Family, marriage and other related counsellors*. This latter category includes the job title *addictions counsellor*, whereas the code 4212 includes *Addictions worker*, thus the algorithm-assigned code was considered preferable, being more accurate.

**Table 7:** Performance of Generation 2 compared with Generation 1.

G2^a^	G1^b^				
	No match	One-digit match	Two-digit match	Three-digit match	Four-digit match
No match	N/A^c^	4	2	0	10
One-digit match	3	N/A	0	1	2
Two-digit match	1	0	N/A	1	0
Three-digit match	1	1	0	N/A	1
Four-digit match	5	2	1	0	N/A

^a^G2: Generation 2.

^b^G1: Generation 1.

^c^N/A: not applicable.

### Generation 3

Table 8 lists the unmatched data. Some current job titles were identified as compounds of >1 occupation, although they may match the correct NOC code if run separately using the combination of each part of the job title and the industry title for searching. For Bartender/Waiter, we could use Bartender accommodation and food services to match the NOC code. The algorithm was updated as follows:

After splitting the job title, first search the NOC data by the combination of each job title and the industry title (the combination of the first job title and the industry title has the priority); if the returned data set is empty, then search the NOC data by each job title (the first job title has the priority); if the returned data set is empty, then search the NOC data by the industry title.

**Table 8:** Unmatched data example for Generation 2.

Current job titles	NOC^a^ codes by ACA^b^-NOC	NOC codes manually	Current industries
Retail	0601	6421	Sales
Bartender/waiter	6513	6512	Accommodation and food services

^a^NOC: National Occupational Classification.

^b^ACA: Automated Coding Algorithm.

#### Performance

Compared with the previous algorithm in Table 9, the improvements yielded 4 (0+2+0+2-0-0-0-0) more job titles accurately coded in the fourth position, 4 (2+0+0+0-0-0-0-2+4) more job titles accurately coded in the third position, 3 (0+0+0+0-1-0-0-0+4) more job titles accurately coded in the second position, and 1 (0+0+0+0-0-0-0-2+3) more job title accurately coded in the first position. A total of 4 additional job titles were accurately coded in the fourth position, and no job titles accurately coded in this position were lost.

An example of apparent inaccuracy being overturned by expert review was the job title *Quality Control* with the industry *Food* coded 9465 *Testers and graders, food and beverage processing* by the algorithm. The manual code 2211 *Chemical technologists and technicians* was allocated presumably because of the included job title *quality control technician–food processing*. However, code 9465 includes a wide range of food-related grading and testing jobs, including *quality control checker–food and beverage processing*, and was considered preferable for accuracy because the assumption of technical expertise is not necessary.

**Table 9:** Performance of Generation 3 compared with Generation 2.

G3^a^	G2^b^				
	No match	One-digit match	Two-digit match	Three-digit match	Four-digit match
No match	N/A^c^	0	0	2	0
One-digit match	0	N/A	0	0	2
Two-digit match	1	0	N/A	0	0
Three-digit match	0	0	0	N/A	2
Four-digit match	0	0	0	0	N/A

^a^G3: Generation 3.

^b^G2: Generation 2.

^c^N/A: not applicable.

### Generation 4

By checking the unmatched data in Table 10, it was identified that the term *teacher* was included in the major group title (Textbox 2). The algorithm was updated as follows:

1. Split the industry title and search each title separately; return the NOC data with the highest frequency of the industry title.
2. If >1 NOC code is returned, first filter the results hierarchically by given job title, then filter the results by industry title, and finally filter the results by given job title in the description of the NOC unit group level.

In this manner, the ACA-NOC algorithm respects the hierarchical structure of the NOC (2016) when producing results.

**Table 10:** Unmatched data for Generation 2.

Current job titles	NOC^a^ codes by ACA^b^-NOC	NOC codes manually	Current industries
TEACHER	4021	4032	EDUCATION
LANDSCAPER	2225	2215	LANDSCAPING
Works in fashion show room	5231	5243	Fashion

^a^NOC: National Occupational Classification.

^b^ACA: Automated Coding Algorithm.

**National Occupational Classification (NOC) major group title from NOC (2016) contained teacher.**

- Major Group 40 professional occupations in education services
- 403-secondary and elementary school teachers and educational counsellors
- 4031-secondary school teachers
- 4032–elementary school and kindergarten teachers
- 4033–educational counsellors

#### Performance

Compared with the previous algorithm, the improvements yielded 5 (3+2+5+1-5-0-1-0) more job titles accurately coded in the fourth position, 14 (4+0+6+0-0-0-0-1+5) more job titles accurately coded in the third position, 12 (7+2+0+1-0-1-6-5+14) more job titles accurately coded in the second position, and 16 (9+1+0+0-2-2-0-2+12) more job titles accurately coded in the first position. The difference between the results of ACA-NOC Generation 3 and Generation 4 is shown in Table 11. A total of 11 additional job titles were accurately coded in the fourth position, but 6 job titles accurately coded in this position were lost because of the alphabetical selection order.

The equivalence of an algorithm and manually assigned code that would otherwise be designated as a mismatch was sometimes adjudicated by expert review. The job title *Electrician* with industry given as *Trades* was allocated code 7241 *Electricians (except industrial and power system)* by the algorithm being a two-digit match for the manual code 7202 *Contractors and supervisors, electrical trades and telecommunications occupations*. It was considered on the basis of the information available that either code was acceptable, the main distinction being that for 7202 it includes those who own and operate their own businesses.

**Table 11:** Performance of Generation 4 compared with Generation 3.

G4^a^	G3^b^				
	No match	One-digit match	Two-digit match	Three-digit match	Four-digit match
No match	N/A^c^	9	7	4	3
One-digit match	2	N/A	2	0	2
Two-digit match	0	1	N/A	6	5
Three-digit match	0	0	0	N/A	1
Four-digit match	5	0	1	0	N/A

^a^G4: Generation 4.

^b^G3: Generation 3.

^c^N/A: not applicable.

## Discussion

### Principal Findings

We developed the ACA-NOC algorithm for automatically coding occupation from textual jobs and industry titles. Mannetje and Kromhout [4] discussed the major standard classification systems for occupations and the different methods for coding occupations, such as self-classification, clerical coding, and computer-assisted coding. They identified that the agreement rates for reliable occupation classification with manual coding is 44% to 89% for 4 to 5 digits, 56% to 80% for 3 digits, 61% to 92% for 2 digits, and 75% to 97% for 1 digit. On the basis of the agreement rates mentioned in their study, our method can be considered reliable because the accuracy of our method is in the range of the agreement rates. Overall, we describe the algorithm as rigorous, meaning that it is capable of allowing for spelling and typing errors as well as adjusting for inserted symbols such as hyphenation, forward slash, or punctuation in the search terms. It uses 7 search strategies sequentially to match job titles with the NOC codes. Through iterative development in successive generations of the algorithm, we have incorporated changes that enhance the ability to handle variations in the quality of input data. Additionally, we built a filtering mechanism that respects the hierarchical structure of the coding scheme in code selection. The system outputs a spreadsheet that includes the best-matched NOC codes for a given input, the type of search that found the match, for example, exact match, as well as the list of candidate matches from which the filter algorithm selected the best match. These are available for algorithm developers investigating mismatches on a benchmarking data set.

During development, we found that reviewing apparent coding mismatches was helpful in identifying opportunities for algorithm improvement and in determining that some automatically assigned codes were preferable to those allocated manually. We further tested the ACA-NOC algorithm with 2 other manually coded data sets of 218 and 186 cases, derived from the same question sets and answered by 2 different populations, with production rates and accuracy at least as good as for our development data.

After comparison with manual codes and with expert review, the accuracy of the algorithm is 58.7% at the four-digit level, 65.0% at the three-digit level, 72.3% at the two-digit level, and 81.6% at the one-digit level. We compared the performance of our approach side-by-side with the available web service algorithm coding with the NOC, gaining a 10% improvement with these early efforts. It is also possible to make some comparisons on the basis of algorithms performing the same task, although to different classification systems. A recent study, similarly reporting performance at each digit level of the occupation code, was conducted by Nahoomi [8], who measured the performance of multiple machine learning models (SVM, maximum entropy, and CNN) for coding job titles to the SOC hierarchy. The accuracy did not exceed 56% in the fourth level, 59% in the third level, 67% in the second level, and 77% in the first level. Our results are comparable, although marginally higher at each level.

Taking a somewhat broader comparison with other automated coding systems, highlighted in the introduction, higher accuracy levels at the four-digit level have been reported. These cases, involving other occupation classification systems, have relied on very large sets of training data and up to 3000 rules in hybrid rule–based systems. Investigation into the design of these systems will give us further insights to improve the system we have developed for coding to the NOC. Further pragmatic considerations for direct comparison of systems include (1) the availability of manually curated test sets for performance benchmarking tasks, (2) the quality of input data, which is not only *very short text* but also full of spelling errors and irregularities, (3) design limitations of techniques used, and (4) the speed at which these algorithms can run on dedicated hardware platforms. Given that such information is not readily available, our comparisons are limited in scope.

Nonetheless, given the relatively modest scale of our project, the results are promising, particularly as there are no other available automated coding algorithms for the NOC (2016). We are currently negotiating access to larger sets of patient data manually coded to the NOC for further benchmarking.

Even at the current level of accuracy, significant time and cost savings are possible over manual coding by insurers or researchers in Canada. As identified by Burstyn et al [9], it can take manual coders days to months to code a few 100 occupations to a classification. They reported that it can take 2 months to code approximately 1600 free text descriptors of lifetime occupational histories to the 2010 SOC. The ACA-NOC algorithm will clearly be useful for efficient and cost-effective coding of data sets, allowing human expertise to be more focused on any residual uncoded cases and those with low confidence matches. With a very high production rate, a coding accuracy of approximately 60% to 4 digits, and being comparable with the intersubject performance of human coders [4], our software is both practicable and economically significant, particularly with large data sets becoming available from population health studies in Canada. One such study is the Canadian Partnership for Tomorrow Project, which has occupation information on >300,000 Canadian participants [12]. Additionally, the ACA-NOC may have utility for Workers’ Compensation Boards and other insurers who collate occupational data.

## Abbreviations

ACA: Automated Coding Algorithm
CNN: convolutional neural network
NAICS: North American Industrial Classification
NOC: National Occupational Classification
SOC: Standard Occupational Classification
SVM: support vector machine
